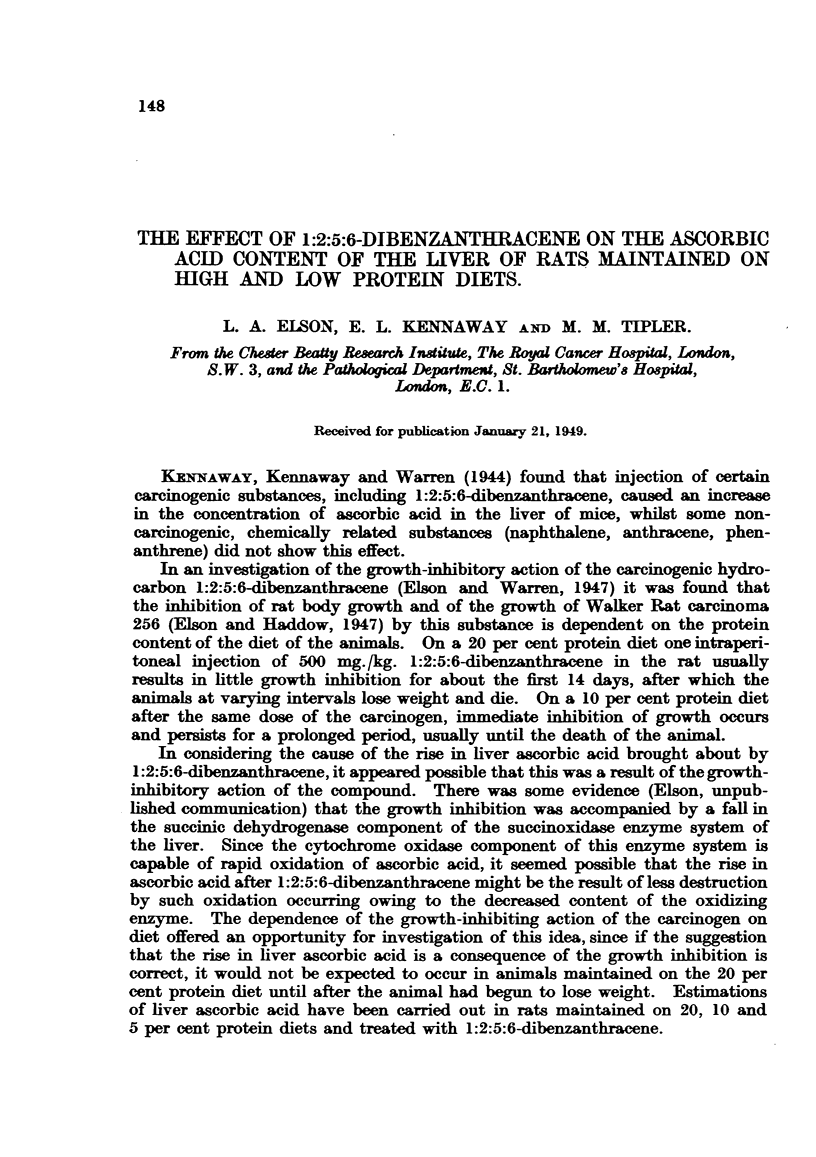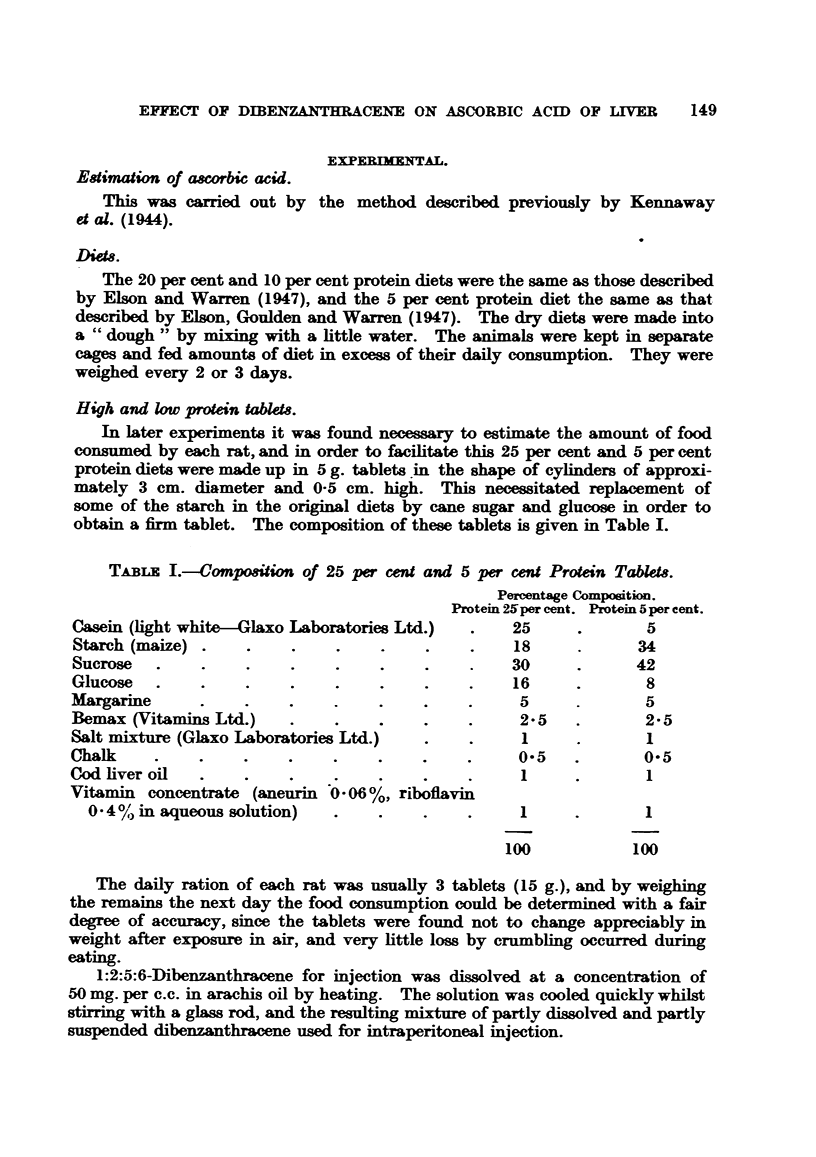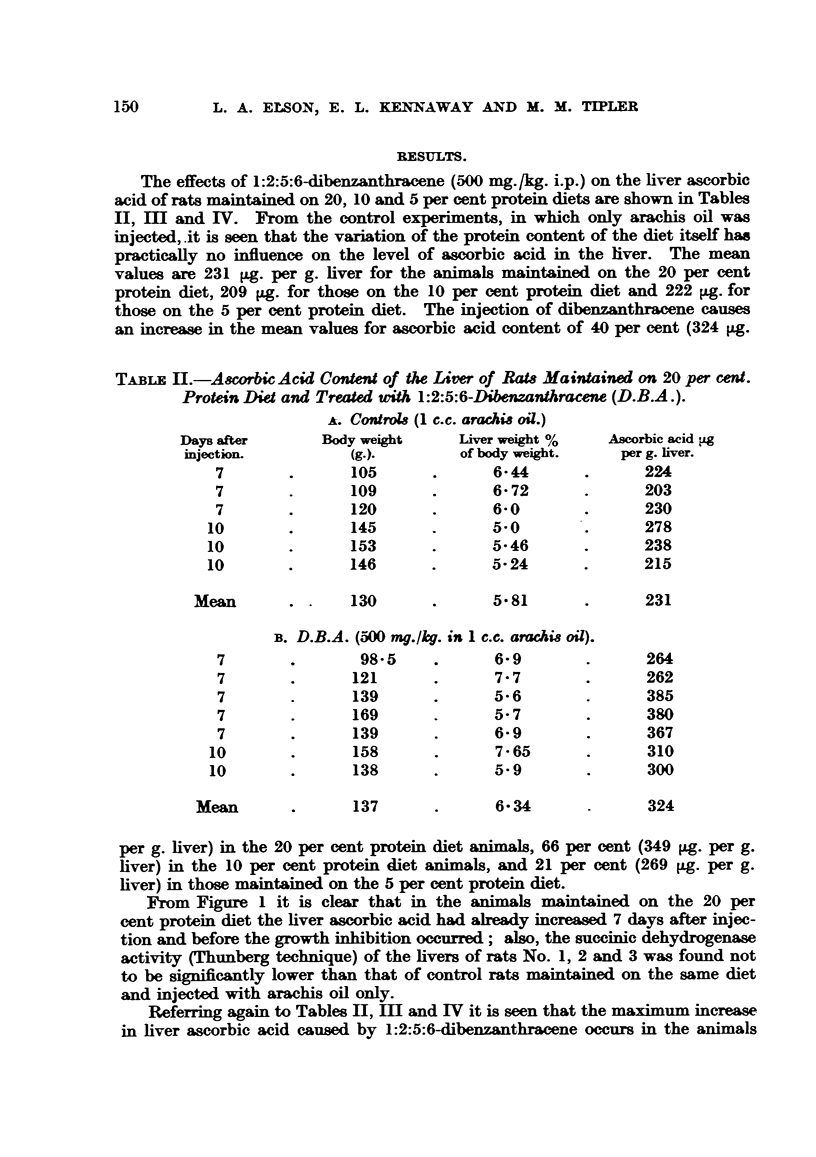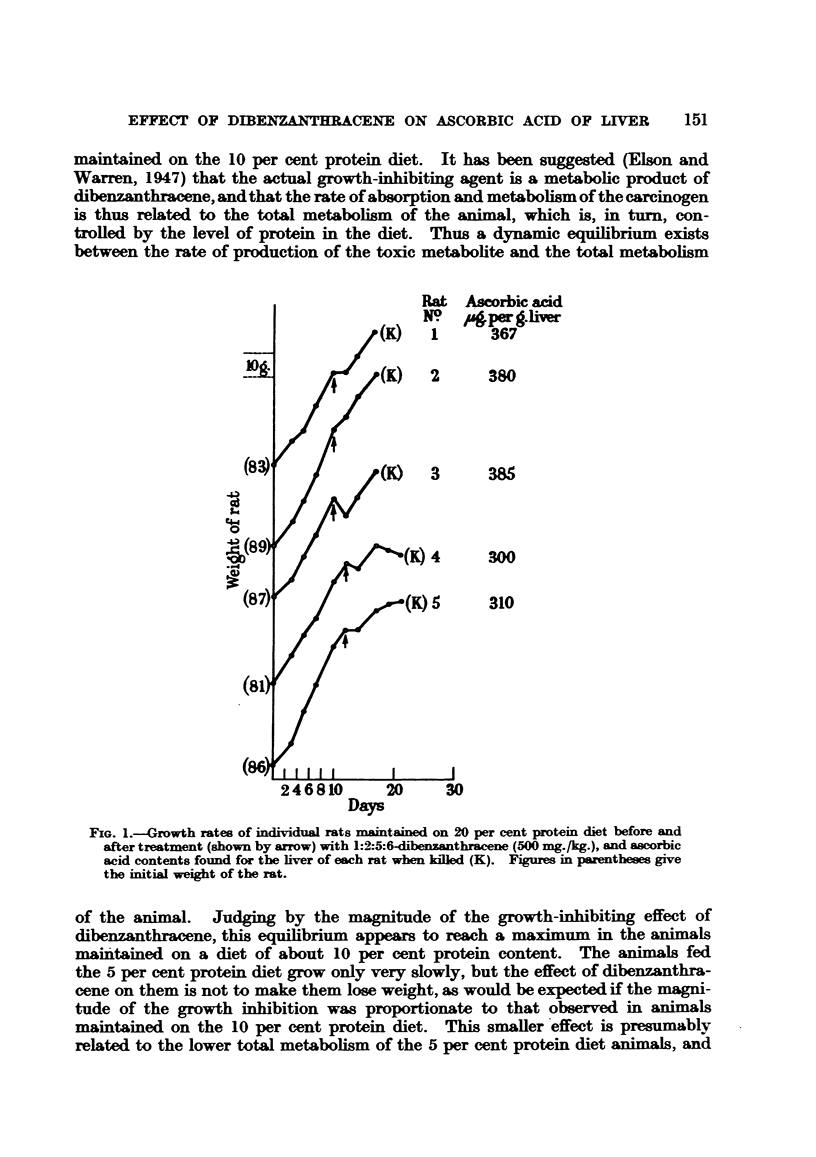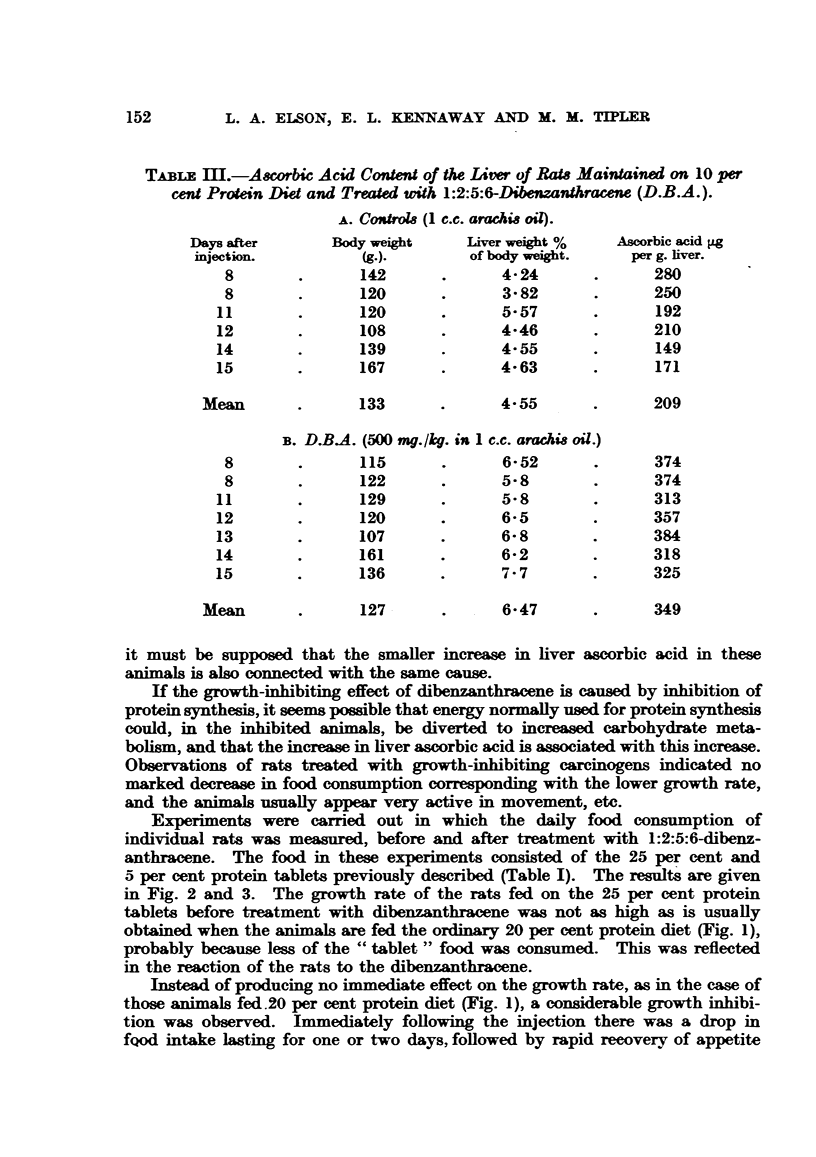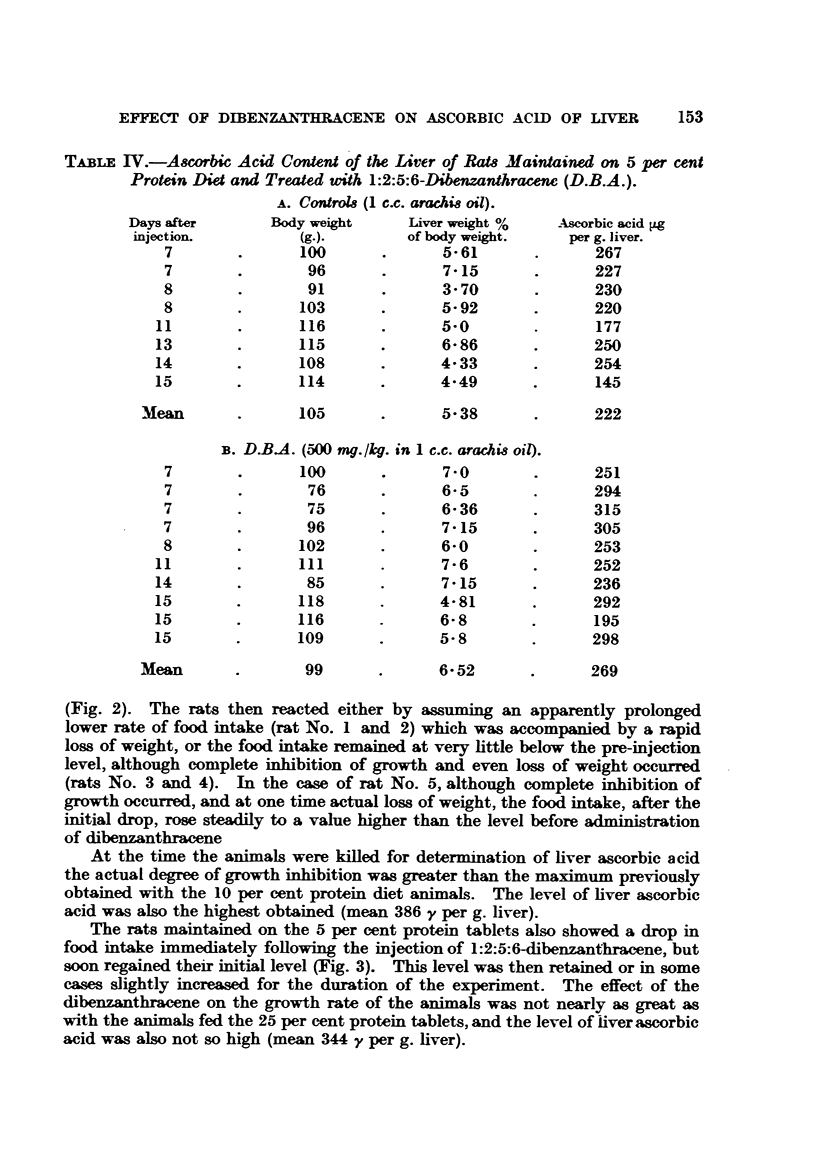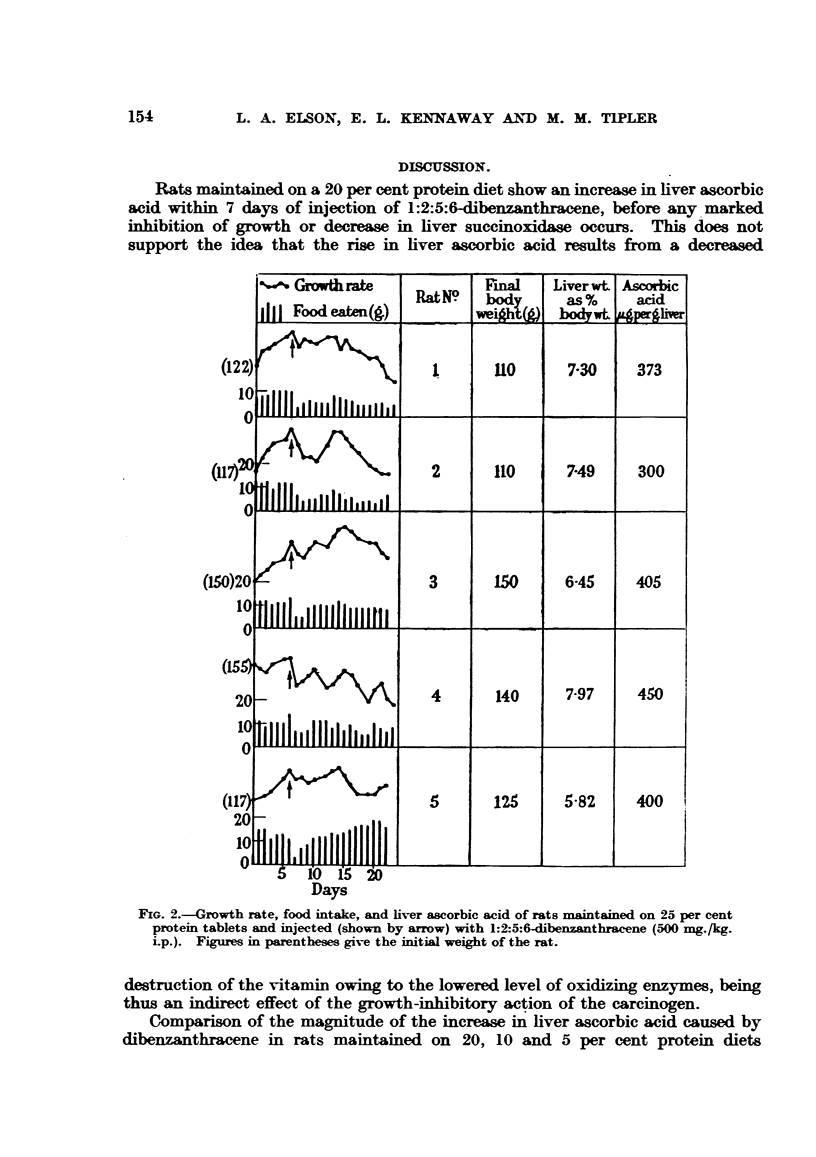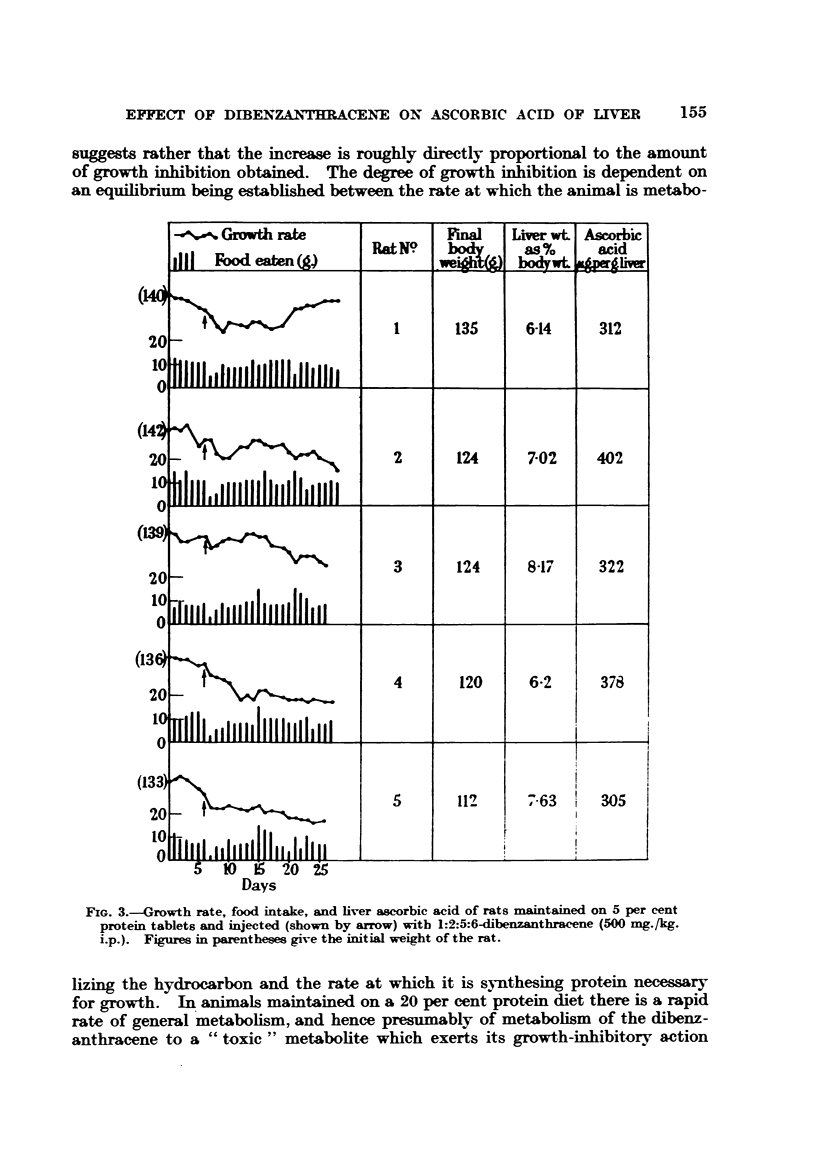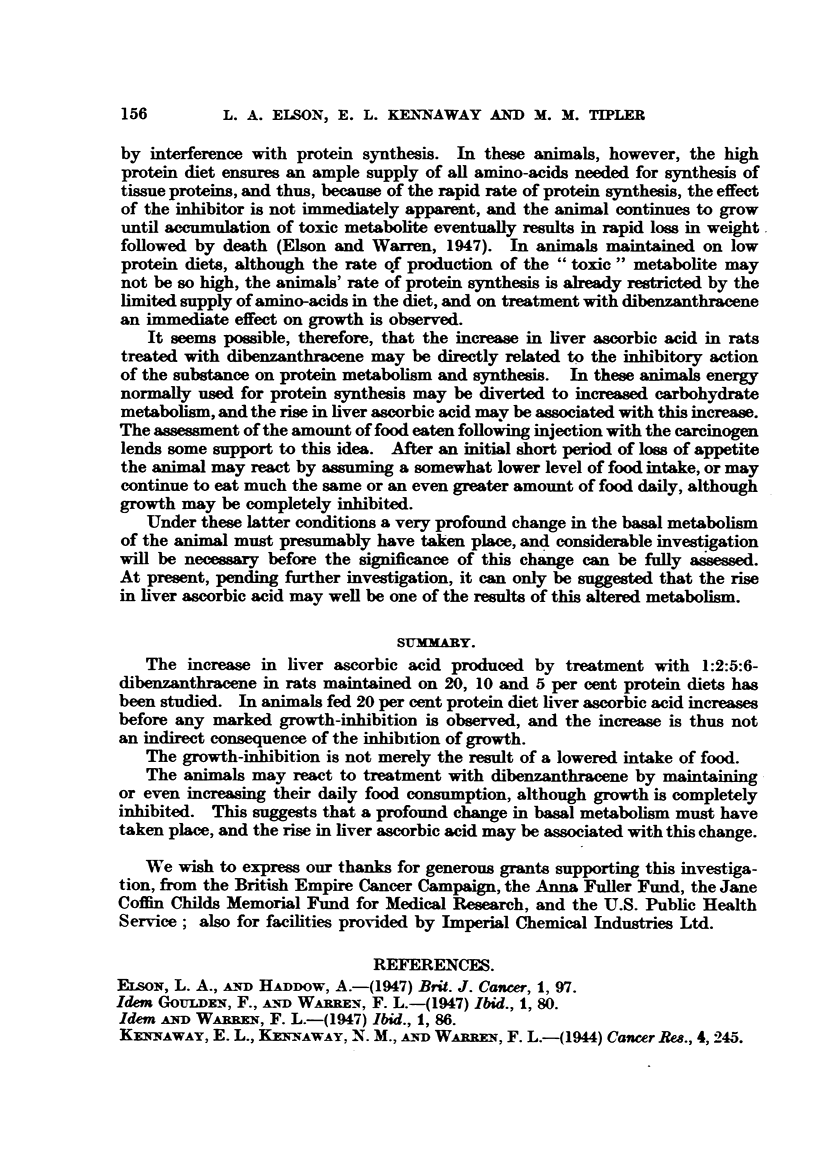# The Effect of 1:2:5:6-Dibenzanthracene on the Ascorbic Acid Content of the Liver of Rats Maintained on High and Low Protein Diets

**DOI:** 10.1038/bjc.1949.17

**Published:** 1949-03

**Authors:** L. A. Elson, E. L. Kennaway, M. M. Tipler


### THE EFFECT OF 1:2:5:6-DIBENZANTHRACENE ON THE ASCORBIC ACID CONTENT OF THE LIVER OF RATS MAINTAINED ON HIGH AND LOW PROTEIN DIETS.

L. A. ELSON, E. L. KENNAWAY AND M. M. TIPLER.

*From the Chester Beatty Research Institute, The Royal Cancer Hospital, London, S.W. 3, and the Pathological Department, St. Bartholomew's Hospital, London, E.C. 1.*

Received for publication January 21, 1949.

KENNAWAY, Kennaway and Warren (1944) found that injection of certain carcinogenic substances, including 1:2:5:6-dibenzanthracene, caused an increase in the concentration of ascorbic acid in the liver of mice, whilst some non-carcinogenic, chemically related substances (naphthalene, anthracene, phenanthrene) did not show this effect.

In an investigation of the growth-inhibitory action of the carcinogenic hydrocarbon 1:2:5:6-dibenzanthracene (Elson and Warren, 1947) it was found that the inhibition of rat body growth and of the growth of Walker Rat carcinoma 256 (Elson and Haddow, 1947) by this substance is dependent on the protein content of the diet of the animals. On a 20 per cent protein diet one intraperitoneal injection of 500 mg./kg. 1:2:5:6-dibenzanthracene in the rat usually results in little growth inhibition for about the first 14 days, after which the animals at varying intervals lose weight and die. On a 10 per cent protein diet after the same dose of the carcinogen, immediate inhibition of growth occurs and persists for a prolonged period, usually until the death of the animal.

In considering the cause of the rise in liver ascorbic acid brought about by 1:2:5:6-dibenzanthracene, it appeared possible that this was a result of the growth-inhibitory action of the compound. There was some evidence (Elson, unpublished communication) that the growth inhibition was accompanied by a fall in the succinic dehydrogenase component of the succinoxidase enzyme system of the liver. Since the cytochrome oxidase component of this enzyme system is capable of rapid oxidation of ascorbic acid, it seemed possible that the rise in ascorbic acid after 1:2:5:6-dibenzanthracene might be the result of less destruction by such oxidation occurring owing to the decreased content of the oxidizing enzyme. The dependence of the growth-inhibiting action of the carcinogen on diet offered an opportunity for investigation of this idea, since if the suggestion that the rise in liver ascorbic acid is a consequence of the growth inhibition is correct, it would not be expected to occur in animals maintained on the 20 per cent protein diet until after the animal had begun to lose weight. Estimations of liver ascorbic acid have been carried out in rats maintained on 20, 10 and 5 per cent protein diets and treated with 1:2:5:6-dibenzanthracene.

### EXPERIMENTAL.

### *Estimation of ascorbic acid.*

This was carried out by the method described previously by Kennaway *et al.* (1944).

### *Diets.*

The 20 per cent and 10 per cent protein diets were the same as those described by Elson and Warren (1947), and the 5 per cent protein diet the same as that described by Elson, Goulden and Warren (1947). The dry diets were made into a " dough " by mixing with a little water. The animals were kept in separate cages and fed amounts of diet in excess of their daily consumption. They were weighed every 2 or 3 days.

### *High and low protein tablets.*

In later experiments it was found necessary to estimate the amount of food consumed by each rat, and in order to facilitate this 25 per cent and 5 per cent protein diets were made up in 5 g. tablets in the shape of cylinders of approximately 3 cm. diameter and 0·5 cm. high. This necessitated replacement of some of the starch in the original diets by cane sugar and glucose in order to obtain a firm tablet. The composition of these tablets is given in Table I.

TABLE I.—*Composition of 25 per cent and 5 per cent Protein Tablets.*

| | Percentage Composition. | |
|---|---|---|
| | Protein 25 per cent. | Protein 5 per cent. |
| Casein (light white—Glaxo Laboratories Ltd.) | 25 | 5 |
| Starch (maize) | 18 | 34 |
| Sucrose | 30 | 42 |
| Glucose | 16 | 8 |
| Margarine | 5 | 5 |
| Bemax (Vitamins Ltd.) | 2·5 | 2·5 |
| Salt mixture (Glaxo Laboratories Ltd.) | 1 | 1 |
| Chalk | 0·5 | 0·5 |
| Cod liver oil | 1 | 1 |
| Vitamin concentrate (aneurin 0·06%, riboflavin 0·4% in aqueous solution) | 1 | 1 |
| | 100 | 100 |

The daily ration of each rat was usually 3 tablets (15 g.), and by weighing the remains the next day the food consumption could be determined with a fair degree of accuracy, since the tablets were found not to change appreciably in weight after exposure in air, and very little loss by crumbling occurred during eating.

1:2:5:6-Dibenzanthracene for injection was dissolved at a concentration of 50 mg. per c.c. in arachis oil by heating. The solution was cooled quickly whilst stirring with a glass rod, and the resulting mixture of partly dissolved and partly suspended dibenzanthracene used for intraperitoneal injection.

### RESULTS.

The effects of 1:2:5:6-dibenzanthracene (500 mg./kg. i.p.) on the liver ascorbic acid of rats maintained on 20, 10 and 5 per cent protein diets are shown in Tables II, III and IV. From the control experiments, in which only arachis oil was injected, it is seen that the variation of the protein content of the diet itself has practically no influence on the level of ascorbic acid in the liver. The mean values are 231 μg. per g. liver for the animals maintained on the 20 per cent protein diet, 209 μg. for those on the 10 per cent protein diet and 222 μg. for those on the 5 per cent protein diet. The injection of dibenzanthracene causes an increase in the mean values for ascorbic acid content of 40 per cent (324 μg. per g. liver) in the 20 per cent protein diet animals, 66 per cent (349 μg. per g. liver) in the 10 per cent protein diet animals, and 21 per cent (269 μg. per g. liver) in those maintained on the 5 per cent protein diet.

TABLE II.—*Ascorbic Acid Content of the Liver of Rats Maintained on* 20 *per cent. Protein Diet and Treated with* 1:2:5:6-*Dibenzanthracene* (*D.B.A.*).

A. *Controls* (1 *c.c. arachis oil.*)

| Days after injection. | Body weight (g.). | Liver weight % of body weight. | Ascorbic acid μg per g. liver. |
|---|---|---|---|
| 7 | 105 | 6·44 | 224 |
| 7 | 109 | 6·72 | 203 |
| 7 | 120 | 6·0 | 230 |
| 10 | 145 | 5·0 | 278 |
| 10 | 153 | 5·46 | 238 |
| 10 | 146 | 5·24 | 215 |
| Mean | 130 | 5·81 | 231 |

B. *D.B.A.* (500 *mg./kg. in* 1 *c.c. arachis oil*).

| Days after injection. | Body weight (g.). | Liver weight % of body weight. | Ascorbic acid μg per g. liver. |
|---|---|---|---|
| 7 | 98·5 | 6·9 | 264 |
| 7 | 121 | 7·7 | 262 |
| 7 | 139 | 5·6 | 385 |
| 7 | 169 | 5·7 | 380 |
| 7 | 139 | 6·9 | 367 |
| 10 | 158 | 7·65 | 310 |
| 10 | 138 | 5·9 | 300 |
| Mean | 137 | 6·34 | 324 |

From Figure 1 it is clear that in the animals maintained on the 20 per cent protein diet the liver ascorbic acid had already increased 7 days after injection and before the growth inhibition occurred; also, the succinic dehydrogenase activity (Thunberg technique) of the livers of rats No. 1, 2 and 3 was found not to be significantly lower than that of control rats maintained on the same diet and injected with arachis oil only.

Referring again to Tables II, III and IV it is seen that the maximum increase in liver ascorbic acid caused by 1:2:5:6-dibenzanthracene occurs in the animals maintained on the 10 per cent protein diet. It has been suggested (Elson and Warren, 1947) that the actual growth-inhibiting agent is a metabolic product of dibenzanthracene, and that the rate of absorption and metabolism of the carcinogen is thus related to the total metabolism of the animal, which is, in turn, controlled by the level of protein in the diet. Thus a dynamic equilibrium exists between the rate of production of the toxic metabolite and the total metabolism

FIG. 1.—Growth rates of individual rats maintained on 20 per cent protein diet before and after treatment (shown by arrow) with 1:2:5:6-dibenzanthracene (500 mg./kg.), and ascorbic acid contents found for the liver of each rat when killed (K). Figures in parentheses give the initial weight of the rat.

of the animal. Judging by the magnitude of the growth-inhibiting effect of dibenzanthracene, this equilibrium appears to reach a maximum in the animals maintained on a diet of about 10 per cent protein content. The animals fed the 5 per cent protein diet grow only very slowly, but the effect of dibenzanthracene on them is not to make them lose weight, as would be expected if the magnitude of the growth inhibition was proportionate to that observed in animals maintained on the 10 per cent protein diet. This smaller effect is presumably related to the lower total metabolism of the 5 per cent protein diet animals, and

TABLE III.—*Ascorbic Acid Content of the Liver of Rats Maintained on* 10 *per cent Protein Diet and Treated with* 1:2:5:6-*Dibenzanthracene* (*D.B.A.*).

A. *Controls* (1 *c.c. arachis oil*).

| Days after injection. | Body weight (g.). | Liver weight % of body weight. | Ascorbic acid μg per g. liver. |
|---|---|---|---|
| 8 | 142 | 4·24 | 280 |
| 8 | 120 | 3·82 | 250 |
| 11 | 120 | 5·57 | 192 |
| 12 | 108 | 4·46 | 210 |
| 14 | 139 | 4·55 | 149 |
| 15 | 167 | 4·63 | 171 |
| Mean | 133 | 4·55 | 209 |

B. *D.B.A.* (500 *mg./kg. in* 1 *c.c. arachis oil.*)

| Days after injection. | Body weight (g.). | Liver weight % of body weight. | Ascorbic acid μg per g. liver. |
|---|---|---|---|
| 8 | 115 | 6·52 | 374 |
| 8 | 122 | 5·8 | 374 |
| 11 | 129 | 5·8 | 313 |
| 12 | 120 | 6·5 | 357 |
| 13 | 107 | 6·8 | 384 |
| 14 | 161 | 6·2 | 318 |
| 15 | 136 | 7·7 | 325 |
| Mean | 127 | 6·47 | 349 |

it must be supposed that the smaller increase in liver ascorbic acid in these animals is also connected with the same cause.

If the growth-inhibiting effect of dibenzanthracene is caused by inhibition of protein synthesis, it seems possible that energy normally used for protein synthesis could, in the inhibited animals, be diverted to increased carbohydrate metabolism, and that the increase in liver ascorbic acid is associated with this increase. Observations of rats treated with growth-inhibiting carcinogens indicated no marked decrease in food consumption corresponding with the lower growth rate, and the animals usually appear very active in movement, etc.

Experiments were carried out in which the daily food consumption of individual rats was measured, before and after treatment with 1:2:5:6-dibenzanthracene. The food in these experiments consisted of the 25 per cent and 5 per cent protein tablets previously described (Table I). The results are given in Fig. 2 and 3. The growth rate of the rats fed on the 25 per cent protein tablets before treatment with dibenzanthracene was not as high as is usually obtained when the animals are fed the ordinary 20 per cent protein diet (Fig. 1), probably because less of the " tablet " food was consumed. This was reflected in the reaction of the rats to the dibenzanthracene.

Instead of producing no immediate effect on the growth rate, as in the case of those animals fed 20 per cent protein diet (Fig. 1), a considerable growth inhibition was observed. Immediately following the injection there was a drop in food intake lasting for one or two days, followed by rapid recovery of appetite

TABLE IV.—*Ascorbic Acid Content of the Liver of Rats Maintained on* 5 *per cent Protein Diet and Treated with* 1:2:5:6-*Dibenzanthracene* (*D.B.A.*).

A. *Controls* (1 *c.c. arachis oil*).

| Days after injection. | Body weight (g.). | Liver weight % of body weight. | Ascorbic acid μg per g. liver. |
|---|---|---|---|
| 7 | 100 | 5·61 | 267 |
| 7 | 96 | 7·15 | 227 |
| 8 | 91 | 3·70 | 230 |
| 8 | 103 | 5·92 | 220 |
| 11 | 116 | 5·0 | 177 |
| 13 | 115 | 6·86 | 250 |
| 14 | 108 | 4·33 | 254 |
| 15 | 114 | 4·49 | 145 |
| Mean | 105 | 5·38 | 222 |

B. *D.B.A.* (500 *mg./kg. in* 1 *c.c. arachis oil*).

| Days after injection. | Body weight (g.). | Liver weight % of body weight. | Ascorbic acid μg per g. liver. |
|---|---|---|---|
| 7 | 100 | 7·0 | 251 |
| 7 | 76 | 6·5 | 294 |
| 7 | 75 | 6·36 | 315 |
| 7 | 96 | 7·15 | 305 |
| 8 | 102 | 6·0 | 253 |
| 11 | 111 | 7·6 | 252 |
| 14 | 85 | 7·15 | 236 |
| 15 | 118 | 4·81 | 292 |
| 15 | 116 | 6·8 | 195 |
| 15 | 109 | 5·8 | 298 |
| Mean | 99 | 6·52 | 269 |

(Fig. 2). The rats then reacted either by assuming an apparently prolonged lower rate of food intake (rat No. 1 and 2) which was accompanied by a rapid loss of weight, or the food intake remained at very little below the pre-injection level, although complete inhibition of growth and even loss of weight occurred (rats No. 3 and 4). In the case of rat No. 5, although complete inhibition of growth occurred, and at one time actual loss of weight, the food intake, after the initial drop, rose steadily to a value higher than the level before administration of dibenzanthracene

At the time the animals were killed for determination of liver ascorbic acid the actual degree of growth inhibition was greater than the maximum previously obtained with the 10 per cent protein diet animals. The level of liver ascorbic acid was also the highest obtained (mean 386 γ per g. liver).

The rats maintained on the 5 per cent protein tablets also showed a drop in food intake immediately following the injection of 1:2:5:6-dibenzanthracene, but soon regained their initial level (Fig. 3). This level was then retained or in some cases slightly increased for the duration of the experiment. The effect of the dibenzanthracene on the growth rate of the animals was not nearly as great as with the animals fed the 25 per cent protein tablets, and the level of liver ascorbic acid was also not so high (mean 344 γ per g. liver).

DISCUSSION.

Rats maintained on a 20 per cent protein diet show an increase in liver ascorbic acid within 7 days of injection of 1:2:5:6-dibenzanthracene, before any marked inhibition of growth or decrease in liver succinoxidase occurs. This does not support the idea that the rise in liver ascorbic acid results from a decreased

| Rat No. | Final body weight (g) | Liver wt. as % body wt. | Ascorbic acid μg per g liver |
|---|---|---|---|
| 1 | 110 | 7·30 | 373 |
| 2 | 110 | 7·49 | 300 |
| 3 | 150 | 6·45 | 405 |
| 4 | 140 | 7·97 | 450 |
| 5 | 125 | 5·82 | 400 |

FIG. 2.—Growth rate, food intake, and liver ascorbic acid of rats maintained on 25 per cent protein tablets and injected (shown by arrow) with 1:2:5:6-dibenzanthracene (500 mg./kg. i.p.). Figures in parentheses give the initial weight of the rat.

destruction of the vitamin owing to the lowered level of oxidizing enzymes, being thus an indirect effect of the growth-inhibitory action of the carcinogen.

Comparison of the magnitude of the increase in liver ascorbic acid caused by dibenzanthracene in rats maintained on 20, 10 and 5 per cent protein diets suggests rather that the increase is roughly directly proportional to the amount of growth inhibition obtained. The degree of growth inhibition is dependent on an equilibrium being established between the rate at which the animal is metabo-

| Rat No. | Final body weight (g.) | Liver wt. as % body wt. | Ascorbic acid μg. per g. liver |
|---|---|---|---|
| 1 | 135 | 6·14 | 312 |
| 2 | 124 | 7·02 | 402 |
| 3 | 124 | 8·17 | 322 |
| 4 | 120 | 6·2 | 378 |
| 5 | 112 | 7·63 | 305 |

FIG. 3.—Growth rate, food intake, and liver ascorbic acid of rats maintained on 5 per cent protein tablets and injected (shown by arrow) with 1:2:5:6-dibenzanthracene (500 mg./kg. i.p.). Figures in parentheses give the initial weight of the rat.

lizing the hydrocarbon and the rate at which it is synthesing protein necessary for growth. In animals maintained on a 20 per cent protein diet there is a rapid rate of general metabolism, and hence presumably of metabolism of the dibenzanthracene to a " toxic " metabolite which exerts its growth-inhibitory action by interference with protein synthesis. In these animals, however, the high protein diet ensures an ample supply of all amino-acids needed for synthesis of tissue proteins, and thus, because of the rapid rate of protein synthesis, the effect of the inhibitor is not immediately apparent, and the animal continues to grow until accumulation of toxic metabolite eventually results in rapid loss in weight followed by death (Elson and Warren, 1947). In animals maintained on low protein diets, although the rate of production of the "toxic" metabolite may not be so high, the animals' rate of protein synthesis is already restricted by the limited supply of amino-acids in the diet, and on treatment with dibenzanthracene an immediate effect on growth is observed.

It seems possible, therefore, that the increase in liver ascorbic acid in rats treated with dibenzanthracene may be directly related to the inhibitory action of the substance on protein metabolism and synthesis. In these animals energy normally used for protein synthesis may be diverted to increased carbohydrate metabolism, and the rise in liver ascorbic acid may be associated with this increase. The assessment of the amount of food eaten following injection with the carcinogen lends some support to this idea. After an initial short period of loss of appetite the animal may react by assuming a somewhat lower level of food intake, or may continue to eat much the same or an even greater amount of food daily, although growth may be completely inhibited.

Under these latter conditions a very profound change in the basal metabolism of the animal must presumably have taken place, and considerable investigation will be necessary before the significance of this change can be fully assessed. At present, pending further investigation, it can only be suggested that the rise in liver ascorbic acid may well be one of the results of this altered metabolism.

### SUMMARY.

The increase in liver ascorbic acid produced by treatment with 1:2:5:6-dibenzanthracene in rats maintained on 20, 10 and 5 per cent protein diets has been studied. In animals fed 20 per cent protein diet liver ascorbic acid increases before any marked growth-inhibition is observed, and the increase is thus not an indirect consequence of the inhibition of growth.

The growth-inhibition is not merely the result of a lowered intake of food.

The animals may react to treatment with dibenzanthracene by maintaining or even increasing their daily food consumption, although growth is completely inhibited. This suggests that a profound change in basal metabolism must have taken place, and the rise in liver ascorbic acid may be associated with this change.

We wish to express our thanks for generous grants supporting this investigation, from the British Empire Cancer Campaign, the Anna Fuller Fund, the Jane Coffin Childs Memorial Fund for Medical Research, and the U.S. Public Health Service; also for facilities provided by Imperial Chemical Industries Ltd.

### REFERENCES.

ELSON, L. A., AND HADDOW, A.—(1947) *Brit. J. Cancer*, 1, 97.

*Idem* GOULDEN, F., AND WARREN, F. L.—(1947) *Ibid.*, 1, 80.

*Idem* AND WARREN, F. L.—(1947) *Ibid.*, 1, 86.

KENNAWAY, E. L., KENNAWAY, N. M., AND WARREN, F. L.—(1944) *Cancer Res.*, 4, 245.